# A microfluidic device enabling drug resistance analysis of leukemia cells via coupled dielectrophoretic detection and impedimetric counting

**DOI:** 10.1038/s41598-021-92647-5

**Published:** 2021-06-23

**Authors:** Yağmur Demircan Yalçın, Taylan Berkin Töral, Sertan Sukas, Ender Yıldırım, Özge Zorlu, Ufuk Gündüz, Haluk Külah

**Affiliations:** 1 Electrical and Electronics Engineering Department, Middle East Technical University, Ankara, Turkey; 2 Mikro Biyosistemler A.Ş., Ankara, Turkey; 3 Mechanical Engineering Department, Middle East Technical University, Ankara, Turkey; 4 METU MEMS Center, Ankara, Turkey; 5 Biology Department, Middle East Technical University, Ankara, Turkey; 6 Present Address: Neuro-Nanoscale Engineering, Mechanical Engineering Department, Eindhoven University of Technology, Eindhoven, The Netherlands; 7 Present Address: Mechanical Engineering Department, Microsystems Section, Eindhoven University of Technology, Eindhoven, The Netherlands

**Keywords:** Biotechnology, Oncology

## Abstract

We report the development of a lab-on-a-chip system, that facilitates coupled dielectrophoretic detection (DEP-D) and impedimetric counting (IM-C), for investigating drug resistance in K562 and CCRF-CEM leukemia cells without (immuno) labeling. Two IM-C units were placed upstream and downstream of the DEP-D unit for enumeration, respectively, before and after the cells were treated in DEP-D unit, where the difference in cell count gave the total number of trapped cells based on their DEP characteristics. Conductivity of the running buffer was matched the conductivity of cytoplasm of wild type K562 and CCRF-CEM cells. Results showed that DEP responses of drug resistant and wild type K562 cells were statistically discriminative (at p = 0.05 level) at 200 mS/m buffer conductivity and at 8.6 MHz working frequency of DEP-D unit. For CCRF-CEM cells, conductivity and frequency values were 160 mS/m and 6.2 MHz, respectively. Our approach enabled discrimination of resistant cells in a group by setting up a threshold provided by the conductivity of running buffer. Subsequent selection of drug resistant cells can be applied to investigate variations in gene expressions and occurrence of mutations related to drug resistance.

## Introduction

Multidrug resistance (MDR) is a condition in which cancer cells develop cross-resistance to distinct drugs of a wide variety of structures and functions. Cancer cells can already have primary (de novo) resistance before the application of chemotherapy. Secondary (acquired) resistance, caused by genetic alterations, can develop in response to drug exposure^1–3^. MDR mechanisms are diverse: (i) Dysfunction of drug influx pumps, (ii) enhanced drug efflux, (iii) alterations in drug targets, (iv) development of anti-apoptotic pathways, (v) metabolic drug inactivation, and (vi) genomic alterations^4^. Enhanced activity of drug efflux pump proteins, mostly caused by the overexpression of ATP-binding cassette (ABC) superfamily proteins, is one of the well-identified mechanisms in MDR. P-glycoprotein (P-gp), multidrug resistance associated protein 1 (MRP1), and breast cancer resistance protein (BCRP) are frequently encountered ABC proteins in MDR cancer cell lines and MDR tumor samples^1^.

MDR impedes curative cancer treatment since MDR patients do not respond to chemotherapy. 90% of metastatic cancer related deaths are due to MDR^5,6^. Moreover, half of cancer patients already have MDR before being exposed to chemotherapy (i.e., primary resistance)^7^. Majidinia et al. reviewed strategies to overcome MDR by presenting conventional and novel approaches^8^.The dominant conventional technique is the inhibition of ABC transporters to prevent drug efflux. Although the clinical success of the first- and second-generation inhibitors is low, third-generation inhibitors are promising. The major drawback of these inhibitors is the lack of specificity for targeting MDR. As novel strategies, nanocarriers have the potential to provide required specificity for targeting MDR cells, hence overcoming MDR by suppressing drug efflux proteins, enhancing drug accumulation inside cells, and inducing apoptosis. These types of novel strategies are cell-specific, therefore respond to patient-to-patient variations. To develop efficient techniques to tackle MDR, the detection and examination of MDR cells are crucial^9^ and can be achieved with the detection of overexpression of drug efflux proteins using in vivo imaging techniques, protein assays, immunogenetic analysis, and flow cytometry^9,10^. Radioactive isotopes, substrates of P-gp and MRP1, are injected to patients’ body in in vivo imaging techniques. Radiolabeled MDR proteins in patient body are monitored by single-photon emission computed tomography and positron emission tomography. In vivo imaging techniques are highly accurate and provide functional information about efflux pumps. However, the repetitive administration of isotopes to patients can be harmful during the prognosis of MDR. Due to their possible damage to patients, clinical transition of in vivo imaging techniques is difficult^10^. Although in vitro methods, such as protein assays, immunogenetic analysis, and flow cytometry, are significantly sensitive, they require expensive labeling agents and long assay durations^9^. In addition, these in vitro techniques cannot provide information about protein functions since they are end-point and static^10^. Apart from the examination of drug efflux proteins with in vivo and in vitro assays, drug toxicity testing can be an alternative way to understand drug response of cancer cells. Recent developments in microfluidic technology have enabled effective drug testing platforms^11,12^. Khamenehfar et al. identified leukemia cells in clinical blood samples by using dielectrophoresis (DEP), i.e. electrical cell detection technique^13^. They achieved to keep leukemia cells in the predetermined position in microchannel and to apply chemotherapeutic drugs and MDR inhibitors on trapped cells. They observed the effect of drugs and MDR inhibitors by applying fluorescence measurement. These platforms provide real-time drug response of cells^13,14^. Electrical approaches as label-free, rapid, and real-time alternative techniques for the diagnosis of MDR have gained popularity in the last two decades since MDR changes the electrophysiological properties of cancer cells. Such methods are electrical cell-substrate impedance sensing^15,16^ and dielectrophoresis (DEP)^17–20^.

DEP is based on generating nonhomogeneous electrical fields to produce forces on particles for their manipulation, where the magnitude of these forces is determined by the dielectric properties of the particles and the running buffer in which particles are immersed^21^. DEP-based MDR studies, which are summarized in Table 1, have been started in 2003 by Labeed et al. They reported that the cytoplasmic conductivity of doxorubicin resistant K562 cells was 2.2 times higher than that of wild type K562 cells, as analyzed by DEP collection spectra^17^. Coley et al. reported that MCF7 cells and their MDR progenies (MCF7-TaxR, MCF7-DoxR, and MCF7-MDR1) had different cytoplasmic conductivities (MCF7-TaxR < MCF7 < MCF7-MDR1 < MCF7-DoxR)^19^. In 2008, Duncan et al. applied ion channel blockers to drug resistant and wild type K562 cells to understand reasons behind differences in cytoplasmic conductivity. As a result, they interpreted that P-gp can have a chloride channel modulation function in cells^22^. All of these studies showed the usability of DEP methods for MDR detection.

**Table 1:** Studies related to DEP-based analysis of MDR cancer cells in terms of cell type, DEP buffer conductivity, the aim of DEP usage (i.e. characterization of cell electrical properties or detection of MDR cells), and quantification method (*LCB* low conductivity buffer, *HCB* high conductivity buffer, *σ* conductivity in mS/m).

Study	Cell type	Buffer, (σ)	DEP characterization	DEP detection	Quantification method
^17^	K562/wt, K562/doxR	LCB, (2.5)	Yes	No	Optical, no staining
^19^	MCF7/wt, MCF7/taxR, MCF7/doxR, MCF7/MDR1	LCB, (2.5)	Yes	No	Optical, no staining
^22^	K562/wt, K562/doxR	LCB, (2.5)	Yes	No	Optical, no staining
^20^	K562/doxR (100 nM), (300 nM), (500 nM), (1000 nM)	LCB, (2.5)	Yes	No	Optical, fluorescent staining
^18^	K562/wt, K562/doxR, K562/imaR	LCB, (2.5)	No	Yes	Optical, fluorescent staining
This study	K562/wt, K562/imaR, CCRF-CEM/wt, CCRF-CEM/doxR	Relatively HCB, (110, 125, 160, and 200)	Yes	Yes	Impedimetric, no staining

We previously reported that the DEP response of MDR cancer cells changed with the level of resistance, i.e. the amount of drug concentration at which cells can survive with continuous drug exposure in their cell growth media by showing resistance to this drug concentration. When the resistance level increased from 100 to 1000 nM doxorubicin in K562 cells, the trapped number of doxorubicin resistant K562 cells on electrodes by DEP increased with a nonlinear trend. The number of trapped cells doubled when the drug resistance level was increased from 100 to 300 nM, while trapped cell number did not linearly rise when the drug resistance level increased to 500 and 1000 nM. This nonlinear relationship inferred that P-gp overexpression may not be reason for MDR at high drug resistance levels or cell cytoplasmic conductivity did not increase with an increase in P-gp expression^20^. We also reported that the detection of imatinib (500 nM) and doxorubicin (500 nM) resistant K562 cells in a mixture, composed of MDR and wild type cells, was achievable by an isolated 3D-electrode DEP device^18^. MDR cells were trapped on electrodes by positive DEP (pDEP) at the frequency, which was chosen to apply no DEP force for wild type cells according to electrical modeling of cells. Low conductivity buffer (LCB) was used to obtain enough polarity for the manipulation of cells (Table 1). However, LCBs alter the electrical properties of cells due to the ion leakage. Gascoyne et al. reported that the DEP response of cells may be affected in LCBs in a short duration (~ 15 min) due to ion leakage^23^. Sabuncu et al. showed that this duration changed from cell to cell. They showed that chondrocytes had comparatively constant crossover frequency (*f*_*cross*_), i.e. the frequency at which DEP force is theoretically equal to zero, values while that of Jurkat cells decreased over time throughout the same analysis duration in an LCB^24^.

In this study, a DEP detection (DEP-D) unit was coupled to impedimetric counting (IM-C) units in a lab-on-a-chip (LOC) system. Two IM-C units were placed upstream and downstream of the DEP-D unit to count the cells that were trapped in DEP-D unit. Moreover, relatively high conductivity buffers (HCBs), having conductivity values that match the cytoplasmic conductivity of wild type cancer cells, were used to trap MDR cells, which have higher cytoplasmic conductivity than that of HCB, by pDEP. Usage of relatively HCBs built up a threshold value, provided by buffer conductivity, to select MDR cells in the DEP-D unit. This selection provides indirect characterization of cell cytoplasmic conductivity and the detection of MDR cells with respect to the threshold value, created by the conductivity of HCB.

## Materials and methods

### Cell culture, drug resistance development, and drug preparation

K562 (Chronic myeloid leukemia, Prof. Ufuk Gündüz Laboratory, Turkey) and CCRF-CEM (Acute lymphoid leukemia, DSMZ, Germany) cells were cultivated in 1X RPMI1640 medium (Medsantek/dist. Gibco, TR) enriched with 10% fetal bovine serum (Interlab/dist. BI, TR), 1% nonessential aminoacids (Interlab/dist. Sigma Aldrich, TR), and 1% penicillin/streptomycin (Interlab/dist. Sigma Aldrich, TR) in T25 cell culture flasks. Subculturing and drug resistance development procedures were presented in our previous work^25^. The same cells were used in this study after 4 passages. Based on IC_50_ analyses, K562 cells were classified as high-level laboratory drug resistant models with approximately 60-fold resistance to *imatinib* as compared to wild type K562 cells, while CCRF-CEM cells were categorized as a clinically relevant model with approximately fourfold resistance to *doxorubicin* as compared to their wild type progenies^25,26^.

### LOC system: device design and fabrication

**Figure 1:**
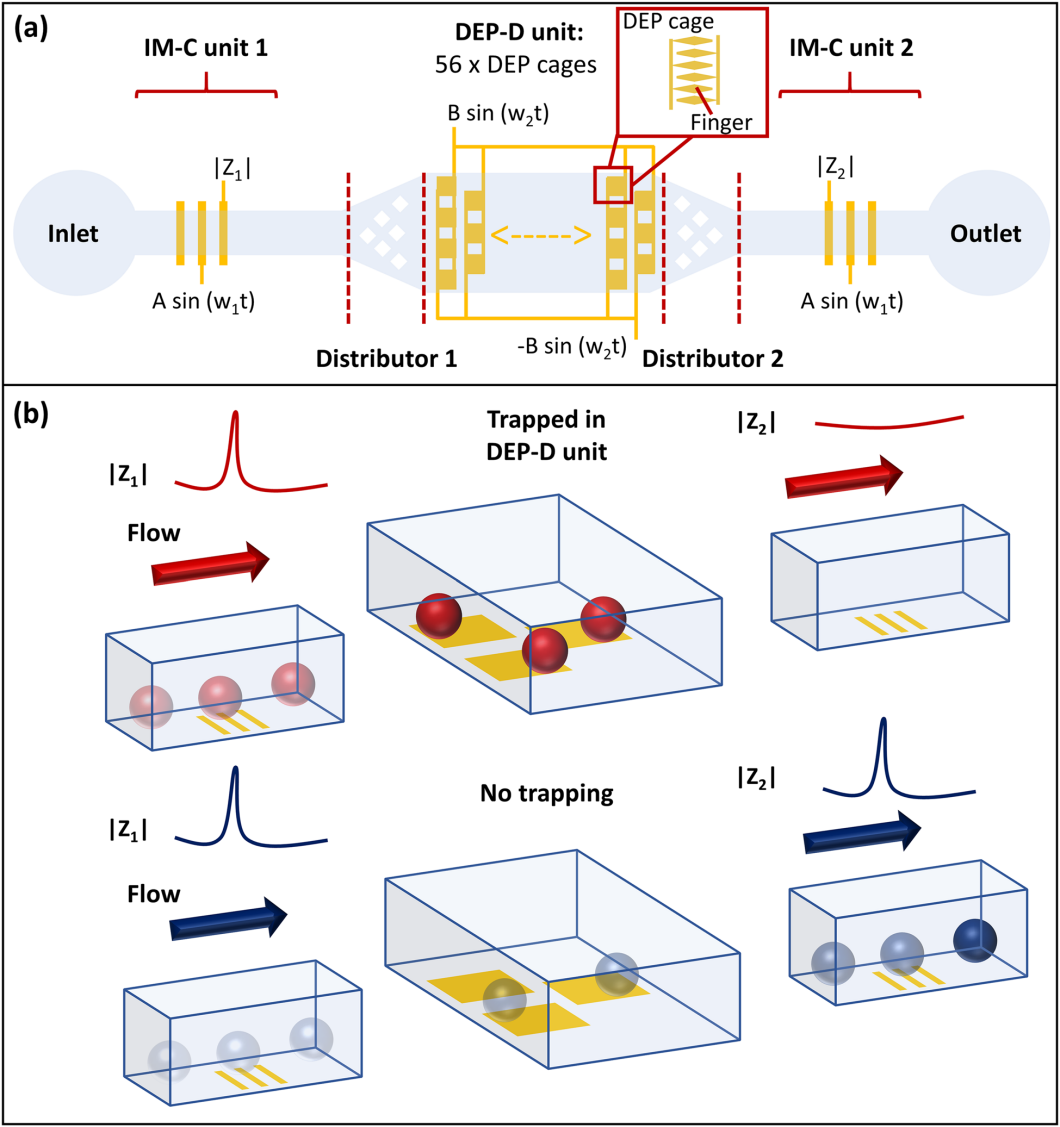
Overall schematic (**a**) and working principle (**b**) of the LOC system, comprising DEP-D and IM-C units.

Figure 1 illustrates the working principle of the LOC system. The LOC system was composed of a single, 25 µm-high parylene microfluidic channel with one inlet and one outlet. One DEP-D unit combined with two IM-C units was located on the channel. In the DEP-D unit (1000 µm-wide), drug resistant cells were differentiated by trapping them on electrodes based on dielectric properties of the cells. Two IM-C units (100 µm-wide) were placed for enumeration before and after trapping. The number of cells trapped in DEP-D unit can be calculated by taking the difference between the number of incoming and outgoing cells. The DEP-D unit had an array of 56 DEP cages (each 120umx120um). Each DEP cage had 6 fingers, composed of diamond-shaped planar electrodes and isolated from the cell solution by a thin parylene layer (~ 0.5 μm). Fingers were aligned such that the minimum distance between two diamond-shaped electrodes was 5 µm. The IM-C units were equipped with 20 µm-wide coplanar excitation and reading electrodes, with 30 µm spacing in between. The excitation and reading electrodes touch to the cell solution. A distributor consisting of diamond-shaped parylene obstacles was placed between DEP-D and IM-C units to enable a homogenous cell distribution across the DEP-D unit.

**Figure 2:**
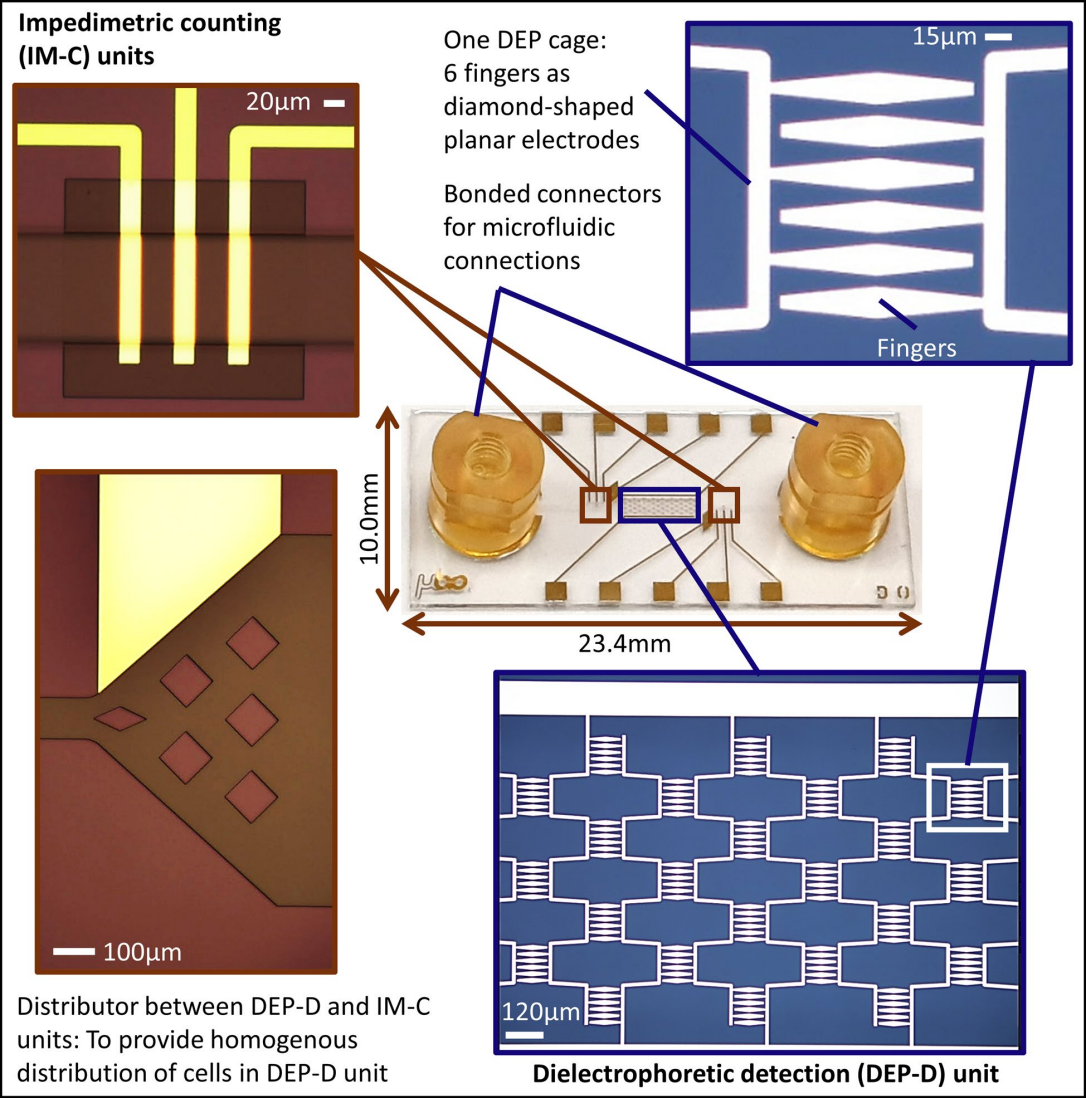
Fabricated LOC system emphasizing electrodes in DEP-D and IM-C units and flow distributor.

LOC device was fabricated on glass wafer with Parylene-C microchannel and gold electrodes for DEP-D and IM-C units by applying sputtering, lithography, and wet etching for gold electrodes, and polymer coating and reactive ion etching steps for microchannel. Details of fabrication is presented in Supplementary material (Sect. 1, Fig. S1). Figure 2 depicts the fabricated device. Following microfabrication, bonded connectors for fluidic interfacing were mounted on the inlet and outlet. Wire bonding was carried out to provide an electrical connection between the LOC and the custom-designed flexible printed circuit board (PCB) (Supplementary material, Sect. 2, Fig. S2).

### LOC system: operation principles

The LOC system is an electrohydrodynamic platform. Cells are trapped in the DEP-D unit under continuous flow with a flow velocity of ${v}_{m}$ in the microchannel, implying a maximum condition of ${v}_{m}$ that is determined by the fact that the DEP force should be greater than the drag force to trap a cell in a DEP cage. This maximum velocity condition is expressed in the relation below^27^. The derivation of this condition is presented in Supplementary material (Sect. 3).

1 $$v_{m} < \frac{{\varepsilon _{m} r^{2} \text{Re} \left( {f_{{CM}} } \right)\nabla \left| E \right|^{2} }}{{3\mu }}$$

In this inequality, ${v}_{m}$*,* $\mathrm{\mu }$, and ${\mathrm{\varepsilon }}_{m}$ are the flow velocity, viscosity, and permittivity of the medium (i.e., buffer) in which the cell is immersed, respectively. $r$ is cell radius. $\nabla {|E|}^{2}$ states the gradient of the external electric field magnitude square, which depends on the electrode and the microchannel geometry, and applied voltage characteristics. $\mathrm{R}\mathrm{e}({f}_{CM})$ is the real part of the Clausius–Mossotti factor, which depends on the frequency, and dielectric properties of the cell and the buffer^28^. Dielectric properties of the cell are not controllable. Therefore, for LOC system operation, buffer, voltage, and flow properties should be appropriately selected to trap the cells considering the condition given in Eq. (1).

#### Selection of buffer conductivity and working frequency of the DEP-D unit

During typical practice, cells are attracted towards a stronger electric field by positive DEP (pDEP), or they are repelled towards a weaker electric field by negative DEP (nDEP). Theoretically, there is a crossover frequency (*f*_*cross*_) at which DEP force experienced by the cells switches from pDEP to nDEP regimes. At *f*_*cross*_, DEP force is equal to zero since *Re(f*_*CM*_*)* of cells is zero^28^. LCBs are used to see this switching^24,29^. High conductivity buffers (HCBs) are defined as buffers having conductivity higher than that of cell cytoplasm^24^. When HCBs, such as phosphate buffered saline (PBS, σ = 1500 mS/m for 1X concentration^30^), are used, only nDEP occurs since cells experience only negative *Re(f*_*CM*_*)* values in HCBs, therefore pDEP is not an option for these buffers. We used relatively HCBs at a conductivity close to the estimated cytoplasmic conductivity of wild type cells (e.g., σ = 200 mS/m) to enable switching between nDEP and pDEP becomes less sensitive to small variations in cell cytoplasmic conductivity. MDR cells having higher cytoplasmic conductivity than that of wild type cancer cells as well as the conductivity of HCB were trapped in DEP cages by pDEP in this LOC system. On the other hand, wild type cells having cytoplasmic conductivity not higher than HCB were not trapped. By this approach, a threshold was created for selection of MDR cells by the conductivity of an HCB. Therefore, the range for working frequencies of DEP-D unit to select MDR cells was enlarged.

**Figure 3:**
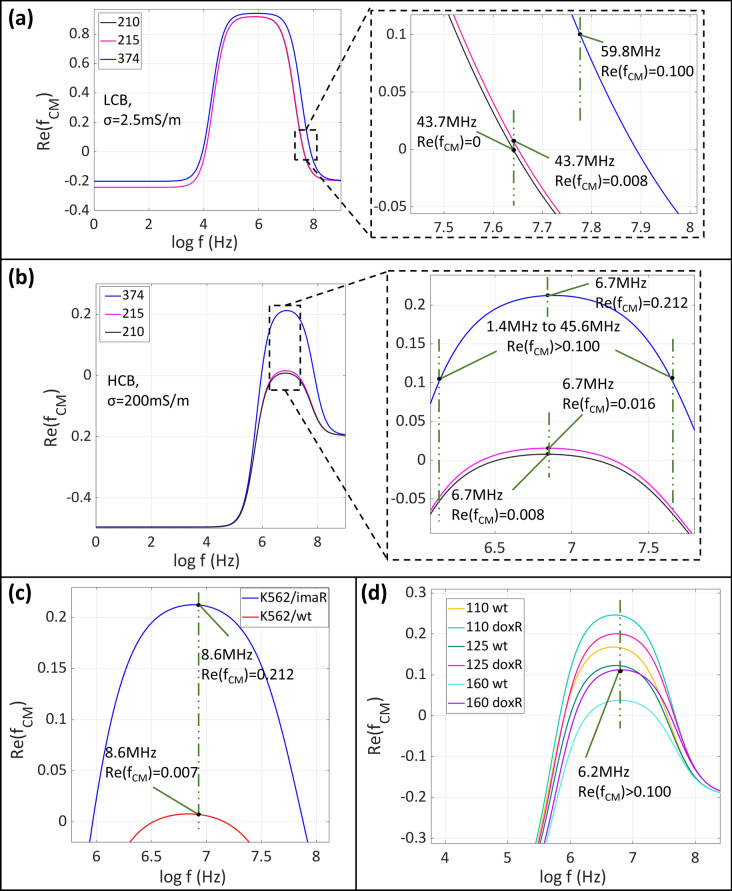
Single shell cell modeling to obtain *Re(f*_*CM*_*)* characteristics of cells having cytoplasmic conductivities of 210, 215, referring K562/wt cells, and 374 mS/m, referring K562/imaR cells, in LCB (conductivity, σ = 2.5 mS/m) (**a**) and relatively HCB (σ = 200 mS/m) (**b**). (**c**) Selection of the working frequency (8.6 MHz) for K562/wt and imaR cells in relatively HCB (σ = 200 mS/m). (**d**) Selection of the working frequency (6.2 MHz) for CCRF-CEM/wt and doxR cells in buffers (at different conductivity values: 110, 125, and 160 mS/m).

To better explain relatively HCB utilization approach, a case study was carried out via MATLAB simulations for *Re(f*_*CM*_*)* of K562/wt and K562/imaR cells. The size and electrical properties of cells, previously obtained by Labeed et al. and Demircan et al.^17,25^ and presented in Supplementary materials (Sect. 4, Table S1), were used in a single shell cell modeling^31^. LCB and HCB had conductivities of 2.5 mS/m and 200 mS/m, respectively. In LCB, *f*_*cross*_ of K562/wt cells, having 210 mS/m cytoplasmic conductivity, was 43.7 MHz (Fig. 3a). When the cytoplasmic conductivity of a cell was changed to 215 mS/m, *Re(f*_*CM*_*)* value became 0.008 at the same frequency. In case 43.7 MHz was chosen as working frequency for obtaining zero *Re(f*_*CM*_*)* for K562/wt cells, a pDEP force could be exerted on a cell instead of zero DEP force for even a 2.4% change in cytoplasmic conductivity. If the buffer velocity, i.e. flow velocity, ${v}_{m},$ is low enough, trapping of wild type cells becomes inevitable at this frequency. To prevent this, higher frequencies can be chosen to stay on the safe side. However, *Re(f*_*CM*_*)* of K562/imaR cells also decreased with increasing frequency (Fig. 3a, blue line). If the buffer velocity is high enough, the trapping of K562/imaR cells becomes impossible, which is not wanted. The frequency range in the case of LCB was determined to be between 43.7 MHz and 59.8MHZ at which K562/imaR cells had *Re(f*_*CM*_*)* values of 0.25 and 0.10, respectively, to trap K562/imaR cells by pDEP. If the same comparison was achieved in a relatively HCB, having conductivity close to the cytoplasmic conductivity of K562/wt cells (σ = 200 mS/m), the frequency interval was defined between 1.4 MHz and 45.6 MHz. Additionally, the increase in *Re(f*_*CM*_*)* of wild type cells was two-fold (from 0.008 to 0.016) and the rate of change in *Re(f*_*CM*_*)* values of both cell types was low with reduced slope in *Re(f*_*CM*_*)* plot (Fig. 3b).

The level of MDR (i.e., high-level laboratory or clinically relevant models) may change the electrophysiological properties of cancer cells^32^. We have previously reported that high-level laboratory MDR model, K562/imaR cells, have 1.8 times higher ion concentration than that of K562/wt cells on average^25^. According to the literature, K562/wt cells have cytoplasmic conductivity in the range of 210–240 mS/m^17^. By using this reference value in the direct relation between cytoplasmic conductivity and ion concentration^33^, the estimated cytoplasmic conductivity values of K562/imaR, CCRF-CEM/wt, and CCRF-CEM/doxR cells were listed in Table 2. The working frequency was selected as 8.6 MHz for K562 cells since at this frequency, K562/imaR cells have maximum *Re(f*_*CM*_*)* while K562/wt cells have the lowest *Re(fCM)* in a buffer at a conductivity of 200 mS/m (Fig. 3c).

**Table 2:** Radii^25^ and estimated cytoplasmic conductivity ranges of K562/wt, K562/imaR, CCRF-CEM/wt, and CCRF-CEM/doxR cells used in the determination of working frequency of the DEP-D unit in HCBs.

Cell type	Cell radius (mean-µm)	Estimated cytoplasmic conductivity ranges (mS/m)	Buffer conductivity (mS/m)	Working frequency (MHz)
K562/wt	5.81	210–240^17^	200	8.6
K562/imaR	6.26	374–427	200	8.6
CCRF-CEM/wt	4.62	185–212	110–125-160	6.2
CCRF-CEM/doxR	5.07	229–261	110–125-160	6.2

In our previous study^25^, no statistically significant difference was reported between the cytoplasmic ion concentration of CCRF-CEM/wt and CCRF-CEM/doxR cells as clinically relevant MDR model. Using our LOC system with appropriate HCB, we hypothesized that if proper buffer conductivity and frequency are determined, statistically different trapping behavior between CCRF-CEM/wt and CCRF-CEM/doxR cells can be obtained. Therefore, the dielectrophoretic responses of CCRF-CEM/wt and CCRF-CEM/doxR cells were examined in 3 different buffers with 110, 125, and 160 mS/m conductivities. The conductivity values were identified based on the minimum expected cytoplasmic conductivity of CCRF-CEM/wt and CCRF-CEM/doxR cells (185 mS/m and 229 mS/m, respectively, Table 2). The frequency was selected as 6.2 MHz, at which CCRF-CEM/doxR cells have maximum *Re(f*_*CM*_*)* value, which ensures the highest pDEP force on resistant cells to trap them on electrodes (Fig. 3d).

#### Flow parameters

Three flow rate values were studied: 10, 5, and 1 µL/min. At 10 and 5 µL/min, trapping was not achieved for any cell type at any of the buffer conductivities and the frequency conditions stated in “LOC system: operation principles”. Therefore, all experiments were carried out at a flow rate of 1 µL/min for all cell types.

#### The working frequency of IM-C unit

The determined buffer conductivity and flow rate conditions remained the same for IM-C units since they were integrated with DEP-D unit. IM-C units were utilized for cell counting. The working frequency was determined as 100 kHz to be able to count cells while DEP-D unit was also under operation with voltages at 6.2 MHz and 8.6 MHz frequencies. Voltage magnitude was determined as 2V_pp_. With voltage values under 2V_pp_, IM-C units did not work due to the interference between IM-C units' excitation signals and the signals used in the DEP-D unit.

#### Cell preparation, experimental, and analysis procedures

Cells were immersed in buffers at different conductivities (presented in Table 2). The procedure was as follows: 1: Washing cells for 3 times with HCB (osmolarity was adjusted to 290 mOsm/L with sucrose and dextrose^34^), where each cycle includes centrifugation (at 300×*g* for 5 min), supernatant removal, resuspension in HCB, consecutively. 2: Trypan blue (1:1, Interlab/ dist. Sigma Aldrich, TR) staining for cell viability check (> 98%) and the determination of final concentration (5 × 10^5^ cells/mL) by an automated cell counter (TC20, Bio-Rad Laboratories Inc.) (Supplementary material, Sect. 5.1, Fig. S3).

The experimental setup is presented in Fig. S4 (Supplementary material, Sect. 5.2). The microchannel was flushed with ethanol and DI water, respectively. To decrease the nonspecific attachment of cells to the parylene surface, poly(l-lysine)-[g]-poly(ethylene glycol) (PLL(20)-g[3.5]-PEG(2), 100 µg/mL in DI water) (SuSoS, Switzerland) coating was applied by treating the device for 1 h at room temperature^35^. Next, the channel was washed with DI water and filled with buffer. Washing were performed at a flow rate of 7.5 µL/min while PLL(20)-g[3.5]-PEG(2) coating and cell analysis were carried out at 1 µL/min by using a coupled pressure generator/mass flow controller with a flow rate sensor (OB1 and BFS, Elveflow, France). DEP-D unit was energized with a signal generator (Agilent, 81150A). Two of its four outputs were utilized at 20V_pp_ with a 180° phase difference. Impedance measurement at IM-C units was performed using an impedance spectroscope (HF2IS, Zurich Instruments, Switzerland) with the application of 2V_PP_ sinusoidal voltage at 100 kHz. Measurement duration was 2 min for each analysis. Nonspecific attachment of cells was determined at the beginning of each experiment while keeping DEP-D unit inactive (i.e., no voltage was applied in DEP-D unit).

Time-based impedance data were collected at IM-C units using HF2IS software (ziControl, Zurich Instruments). We applied baseline removal and peak detection functions in MATLAB to extract cell count information from these data. 100Ω was chosen as the threshold of impedance magnitude for cell detection. In combination with the cell detection algorithm, a cross correlation function, *XCORR*, was also run since there were two different time-dependent sequences: impedance magnitude values obtained in IM-C units at the entrance and outlet of LOC. *XCORR* detected the similarity between IM-C 1 and IM-C 2 and determined the delay in time between them by examining the correlation between two impedance sequences and estimating the time at which maximum correlation occurs^36^. Employing *XCORR*, the time delay between two IM-C units, inherently formed due to the duration that cells spend in the DEP-D unit between IM-C units (Supplementary material, Sect. 5.3, Fig. S5), was identified and eliminated. , This method prevents misinterpretation in the quantification of incoming and outgoing cell counts.

DEP responses of cells were quantified by defining a trapping ratio, which was calculated by the following formula:

2 $$Trapping~\;Ratio = \frac{{incoming~\;cell~\;number - outgoing\;~cell~\;number}}{{incoming\;~cell~\;number}} \times 100$$

### Statistical analysis

Each measurement was repeated with three biological replicates. In order to determine a statistical correlation between these measurements, a one-way ANOVA test with Fisher Least Significant Difference (LSD) to compare means, and Levene’s test for equal variance were utilized. As a result, p values < 0.05 were considered statistically different.

## Results and discussion

Read-out of the LOC system was two impedance spectra (Supplementary material, Sect. 5.3, Fig. S5). Videos S1 and S2 demonstrate cells as impedance peaks to exemplify cell trapping and no cell trapping cases, respectively. The following subsections present the dielectrophoretic behavior of K562 and CCRF-CEM cells and their drug resistant progenies.

### Dielectrophoretic response of K562 cells

**Figure 4:**
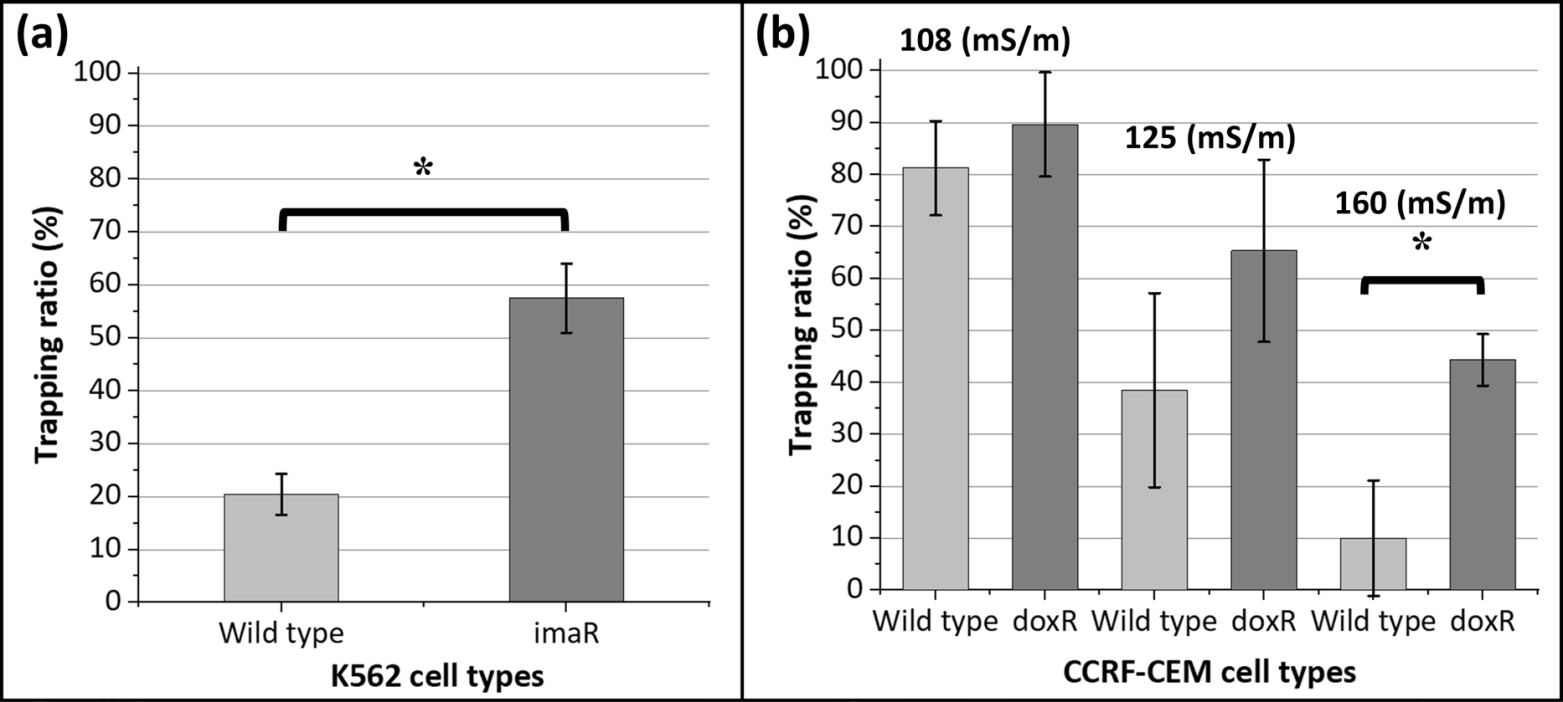
Dielectrophoretic behavior of leukemia cells and their drug resistant progenies. (**a**) The trapping ratio of K562/wt and K562/imaR cells immersed in relatively HCB (σ = 200mS/m) at 8.6 MHz under 20V_pp_ and at a flow rate of 1 µL/min. (**b**) The trapping ratio of CCRF-CEM/wt and CCRF-CEM/doxR cells in different buffers, with 110, 125, and 160 mS/m conductivity, at 6.2 MHz under 20V_pp_ and at a flow rate of 1 µL/min. Results are presented as mean ± stdev. * indicates significantly different results at the p = 0.05 level (n = 3).

High-level laboratory models are used to understand the potential mechanisms of drug toxicity and MDR^26^. K562/imaR cells were high-level laboratory models in this study. The hypothesis, built based on the significant difference between the average cytoplasmic conductivity of K562/imaR and K562/wt cells, was that K562/imaR cells should be trapped on electrodes, while wild type cells should flow to the outlet. By using *Re(f*_*CM*_*)* values and size information of these cells (see Fig. 3c and Table 2), DEP force acting on K562/imaR cells was calculated as 38 times higher than that of K562/wt cells in relatively HCB (σ = 200mS/m) at 8.6 MHz. The calculation is presented in Supplementary material (Sect. 6.1). By applying 20V_pp_ voltage and under 1 µL/min flow rate, the trapping ratios were 20% (± 3.9%) and 58% (± 6.6%) for K562/wt and K562/imaR cells, respectively (Fig. 4a), which had negligible nonspecific trapping ratios (Supplementary material, Sect. 6.2, Table S2). The averages of the trapping ratio of these cells were significantly different (n = 3, p = 0.05). As proof of our hypothesis, high-level laboratory models of MDR and wild type progenies can be discriminated by employing their DEP responses and analyzed in terms of their physiological behaviors. Analysis was repeatable with low standard deviation values (Coefficient of variation values, calculated as 19.5% and 11.4% for K562/wt and K562/imaR, respectively).

One-fifth of K562/wt cells were consistently trapped with three biological replicates. This showed that one-fifth of the K562/wt cell population had higher cytoplasmic conductivity than the conductivity of relatively HCB (σ = 200mS/m). As explained in Fig. 3b, by a 2.4% increase in cytoplasmic conductivity, the DEP force can be doubled due to an increase in *Re(f*_*CM*_*)*. This increase was caused by ion concentration variations in the cytoplasm of these cells. The ion concentration can change with drug response characteristics^22^. Therefore, potentially drug resistant cells in a wild type cell population can be selected by trapping them in the DEP-D unit without applying drug exposure prior to analysis, which indicates the detection of primary type drug resistance. The utilization of relatively HCB created a thresholding mechanism with respect to cytoplasmic conductivity of cells. This can be used as a subpopulation detection method.

The trapping ratio of K562/imaR cells was almost 60%. This means that 40% of K562/imaR cells were not trapped. This was expected since not all resistant cells had high enough cytoplasmic conductivity to be trapped in a buffer, having 200 mS/m conductivity. As we mentioned before, cytoplasmic conductivity was also dependent on the drug resistance level. The drug resistance level was not constant in this population, which is the inherent property of a resistant cell line. Therefore, when determining the drug resistance level via cytotoxicity assays, 50% of the population's vitality is the norm^37^. This means that the other half is death under a predefined amount of drug exposure. Therefore, with a standard deviation value of 6.6%, 58% trapping was in accordance with the cytotoxicity results of this model (IC_50_ values, presented in^25^), indicating the detection of acquired (secondary) resistance.

Flow cytometry results showed that the differences in dielectrophoretic response, which is an indicator of cells' drug response characteristics, can be caused by the variation in P-gp expression. There was no difference between the MRP1 and BCRP protein levels of K562/wt and K562/imaR cells as P-gp level were 2.5 times higher in K562/imaR cells (See Supplementary materials Sect. 7 and Fig. S6 for details of flow cytometry analysis). Flow cytometry results also displayed that K562/wt cells had P-gp expression at a level that cannot be neglected although it was lower than that of K562/imaR cells. There are evidences in the literature that P-gp can be intrinsically expressed in chronic lymphocytic leukemia cells^38^ and K562 cells^39^, which is a chronic myelogenous leukemia model with lymphoblastic morphology^40^. This P-gp phenotype can be the probable cause of the 20% trapping ratio of K562/wt cells by increasing the cytoplasmic ion concentration of K562/wt cells.

### Dielectrophoretic response of CCRF-CEM cells

CCRF-CEM/doxR cells were used as clinically-relevant models in this study^26^. The variation between the average cytoplasmic ion concentration of CCRF-CEM/doxR and CCRF-CEM/wt cells was not significantly different (p < 0.05 level)^25^. This indicated that the cumulative type of analysis was not sensitive enough to detect differences between these cells. Therefore, single-cell behavior was investigated utilizing the LOC system and HCB approach. Using the predicted cell cytoplasmic conductivities of these cells, three different buffers were chosen at 110, 125, and 160 mS/m conductivities and working frequency was determined as 6.2 MHz as explained in Sect. 2.3.1. The nonspecific trapping ratios were not significantly different (p < 0.05 level) for CCRF-CEM/doxR and CCRF-CEM/wt cells at each conductivity, which were neglected (Table S2 in Supplementary material, Sect. 6.2). According to *Re(f*_*CM*_*)* simulations, it was expected that the cell trapping ratio decreases with increasing buffer conductivity. The hypothesis was constructed that the decrease in trapping of CCRF-CEM/wt cells should be more rapid than that of CCRF-CEM/doxR cells for one of these conductivities. The results demonstrated a decrease in the cell trapping ratios (Fig. 4b). At 110 and 125 mS/m conductivities, the trapping regime of these two cell types was not significantly different, although the trapping ratio of CCRF-CEM/doxR cells was higher than that of CCRF-CEM/wt cells in all buffers. A statistically significant difference was obtained for CCRF-CEM/doxR and CCRF-CEM/wt cells in the buffer at a conductivity of 160 mS/m. However, the trapping ratio was decreased to 44.3% (± 4.9%) from 89.7% (± 10.1%) for CCRF-CEM/doxR cells. The CCRF-CEM/doxR cell line is not a stable drug resistance model since it is clinically-relevant^26^. This decrease in the trapping ratio was expected. On the other hand, with a more rapid decrease in the trapping ratio of CCRF-CEM/wt cells from 81% (± 9.1%) to 10.0% (± 11.1%), the dielectrophoretic behavior of these cells was a significant indicator to select drug resistant ones by proving our hypothesis and showing the importance of this single-cell analysis LOC platform.

Flow cytometry results demonstrated a sixfold increase in P-gp expression in CCRF-CEM/doxR cells and no difference between MRP1 and BCRP protein levels compared to CCRF-CEM/wt cells. Wild type CCRF-CEM cells did not show a significant amount of P-gp expression since this cell type is the model of acute lymphoblastic leukemia, and to the best of our knowledge, there is no evidence for P-gp expression in these cells. Additionally, Illmer et al. reported 74-fold less MDR-1 gene product, i.e. a gene used to express P-gp, and a reduced amount of P-gp in CCRF-CEM/wt cells when it was compared with K562/wt cells via real-time polymerase chain reaction (PCR) analysis^39^.

## Conclusion and outlook

MDR is a challenge in chemotherapy since MDR causes the cancer patient not to respond to the treatment. Different mechanisms cause MDR, and the most common one is the increase in drug efflux. Detection methods for MDR cells have been developed based on the physical and functional monitoring of drug efflux pumps. These pumps create electrophysiological differences between MDR and wild type cells by ion channel modulating feature of them. Therefore, electrical approaches in the recognition of MDR cells have gained traction in the last two decades. DEP is one of these electrical approaches. In this study, a DEP-based MDR detection unit was combined with two IM-C units to quantify the drug resistance of leukemia cell lines in a label-free manner. As a novel approach, relatively HCBs were utilized to generate a selectivity threshold for detecting MDR levels in leukemia cells. This novel approach eliminates the necessity of determining a unique crossover frequency for wild type cells, which is challenging since there may be cells with unknown levels of drug resistance in the population, with a relatively high level of heterogeneity.

The results revealed that comparing different cell types and identifying subpopulations in heterogeneous populations could be achieved with high repeatability by utilizing the HCB approach. To make a more elaborated interpretation of the dielectrophoretic behavior of cells in relation to their drug response, downstream genetic analysis with trapped and non-trapped cells should be carried out, including PCR and Western blotting.

## Supplementary Information

Supplementary Information 1.

Supplementary Video 1.

Supplementary Video 2.
