# Supplementary material for: A microfluidic device enabling drug resistance analysis of leukemia cells via coupled dielectrophoretic detection and impedimetric counting
Source: Sci Rep. 2021 Jun 23;11:13193. doi: 10.1038/s41598-021-92647-5 (PMC8222334; doi:10.1038/s41598-021-92647-5)
Supplement: Supplementary file 1 — Supplementary Information 1. [file 41598_2021_92647_MOESM1_ESM.docx]

**SUPPLEMENTARY MATERIAL**

**A microfluidic device enabling drug resistance analysis of leukemia cells via coupled dielectrophoretic detection and impedimetric counting**

Yağmur Demircan Yalçın^1, 2, *^, Taylan Berkin Töral^2^, Sertan Sukas^2, **^, Ender Yıldırım^2, 3^, Özge Zorlu^2^, Ufuk Gündüz^5^, and Haluk Külah^1, 2, 4^

*^1^Middle East Technical University, Electrical and Electronics Engineering Department, Ankara, Turkey*

*^2^* *Mikro Biyosistemler A.Ş., Ankara, Turkey*

*^3^ Middle East Technical University, Mechanical Engineering Department, Ankara, Turkey*

*^4^METU MEMS Center, Ankara, Turkey*

*^5^ Middle East Technical University, Biology Department, Ankara, Turkey*

**Corresponding author: Y. Demircan Yalçın, e-mail address:* [*y.demircan.yalcin@tue.nl*](mailto:y.demircan.yalcin@tue.nl)*, present address: Neuro-Nanoscale Engineering, Mechanical Engineering Depart., Eindhoven University of Technology, Eindhoven, Netherlands.*

***Present address: Mechanical Engineering Department, Microsystems section, Eindhoven University of Technology, Eindhoven, Netherlands.*

1. **Fabrication of LOC system**

First, 6’’ circular glass wafers were cleaned to get rid of organic residues with PIRANHA solution (1:1, H_2_SO_4_: H_2_O_2_) for 30 minutes. Afterwards, BHF (1: 7) (HF: NH_4_F) solution etch was done for 5 min for creating surface roughness. Then, the glass wafers were coated with Ti/Au (30nm / 300nm) with Bestec-1 sputter system. The lithography with electrode mask was done with SPR 220-3 positive photoresist at 3000 rpm spin coat. The patterning was done with metal etch process. The undercut was measured to be approximately 1.0µm at the final step of the metal etch. The metal etch masking photoresist was stripped with PRS 2000 photoresist stripper at 80 ⁰C for 15 minutes in two repetitive tanks. Then, the glass wafers were coated with 0.5µm Parylene-C (measured to be 0.54µm) in polymer coater (SCS PDS 2010, SCS Coating, UK and USA). The parylene insulation layer was patterned by RIE (SPTS RIE system) using the lithographically patterned mask of SPR 220-3 spun at 3000 rpm to open the surface of electrodes in impedimetric counting (IM-C) unit. Afterwards, the microchannel and input/output ports were formed by AZ 40XT photoresist lithography. The microchannel thickness was measured to be 20.1µm (2800 rpm for photoresist spin coating) after the photolithography. Then, the glass wafers were coated with 20µm Parylene-C and measured to be 20.2µm to form microchannel. After this step, the microchannels I/O ports and metal contact pads opening RIE process were carried out. After dicing of devices (49 dices per 6” circular wafer), sacrificial photoresist layer was dissolved in acetone.

**
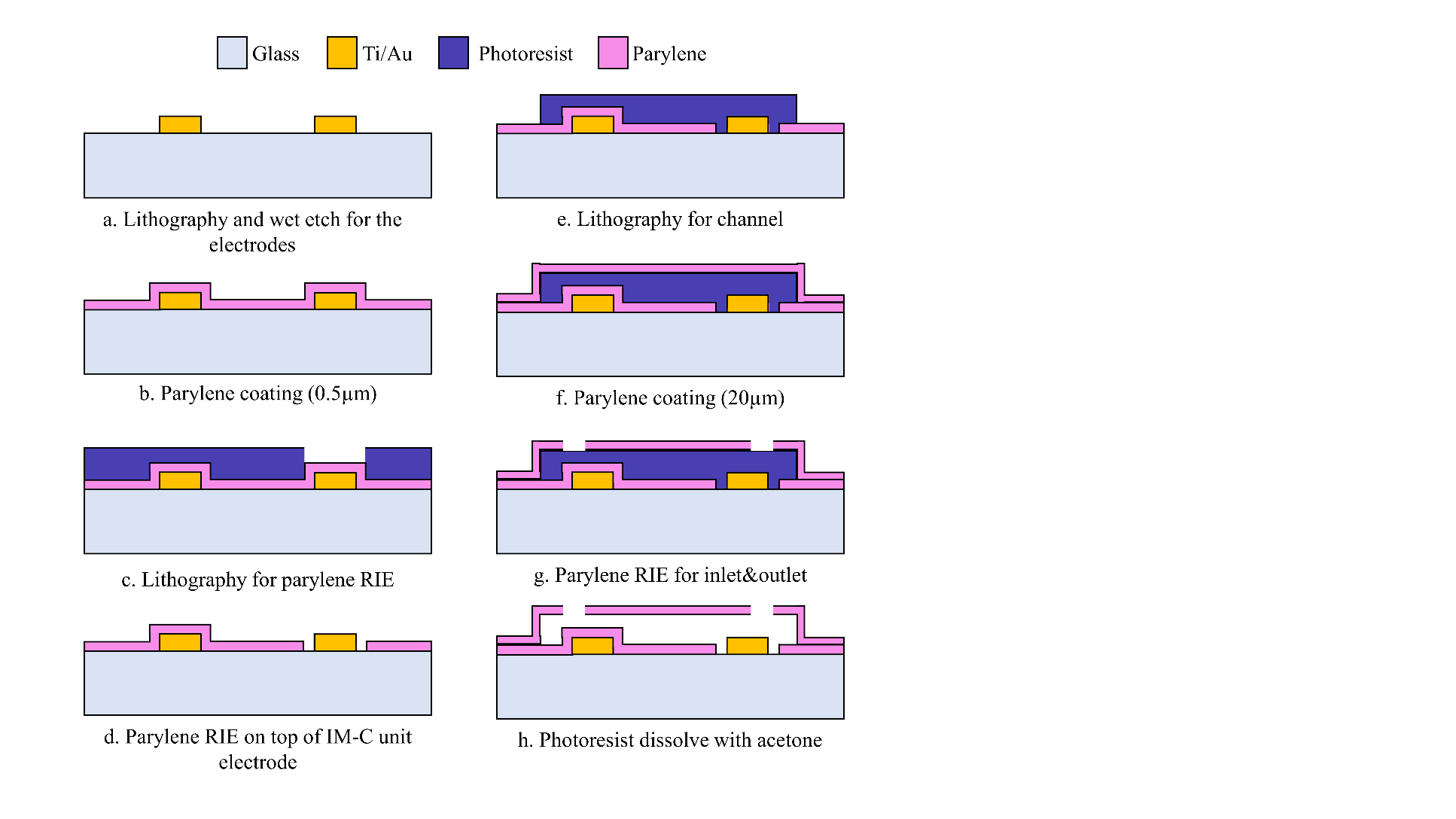
**

Figure S1: Fabrication flow of LOC (left and right side of the images were used as dielectrophoretic detection (DEP-D) unit and IM-C units, respectively).

1. **Custom design flexible PCB**

Lab on a chip (LOC) system was placed on a custom design flexible PCB by epoxy (not presented in image to provide better visualization). Electrical contacts were provided via wire bonding. This PCB was connected to distributor PCB through zero insertion force connector by carrying signals into/from LOC system to/from the signal generator and impedance spectroscope without crosstalk.

Figure S2: Custom design flexible PCB.

1. **Derivation of condition in Equation 1**

DEP force is presented as follows^1^:

| $F_{DEP}=2\pi\varepsilon_{m}r^{3}\mathrm{Re}\left( f_{CM} \right)\boldsymbol{\nabla}{\vert E\vert}^{2}$ | (S1) |
| --- | --- |

$\varepsilon_{m}$ is the medium permittivity. *r* is the cell radius and Re*(f_CM_)* is the real part of Clausius–Mossotti factor of the cell. $\boldsymbol{\nabla}{|E|}^{2}$ states the gradient of the external electric field magnitude square.

Drag force is presented as follows^2^:

| $F_{Drag}=6\pi\mu r(v_{m}-v_{p})$ | (S2) |
| --- | --- |

*v_m_* and *µ* are the velocity and viscosity of medium (i.e. buffer) in which cells are immersed, respectively. *v_p_* is the cell velocity.

To manipulate cells via DEP force under continuous flow, DEP force should be greater than drag force. If a cell is trapped on electrodes under flow, *v_p_* is equal to zero. When these are applied, condition in equation S3 is obtained.

| $6\pi\mu rv_{m}< 2\pi\varepsilon_{m}r^{3}\mathrm{Re}\left( f_{CM} \right)\boldsymbol{\nabla}{\vert E\vert}^{2}$ | (S3) |
| --- | --- |

If simplifying is applied, condition presented in Equation 1 of manuscript is obtained (Eq. S4).

| $v_{m}<\frac{\varepsilon_{m}r^{2}\mathrm{Re}(f_{CM})\boldsymbol{\nabla}{\vert E\vert}^{2}}{3\mu}$ | (S4) |
| --- | --- |

1. **Cell electrical properties**

A custom script was prepared in MATLAB to obtain *Re(f_CM_)* values of cells by using single shell cell modeling^3^.

Table S1: Electrical properties of K562/wt and K562/imaR cells used in single shell cell modeling to obtain *Re(f_CM_)* values in Figure 3 (a)^4,5^.

|  | Cell radius (µm) | Membrane thickness (nm) | Membrane relative permittivity | Membrane conductivity (mS/m) | Cytoplasmic relative permittivity | Cytoplasmic conductivity (mS/m) |
| --- | --- | --- | --- | --- | --- | --- |
| K562/wt | 5.81 | 10 | 8 | 1.8x10^-3^ | 40 | 210 & 215 |
| K562/imaR | 6.26 | 10 | 9 | 2x10^-3^ | 40 | 374 |

1. **Test and analysis procedure**
   1. **Cell preparation**

Figure S3 presents cell preparation steps.


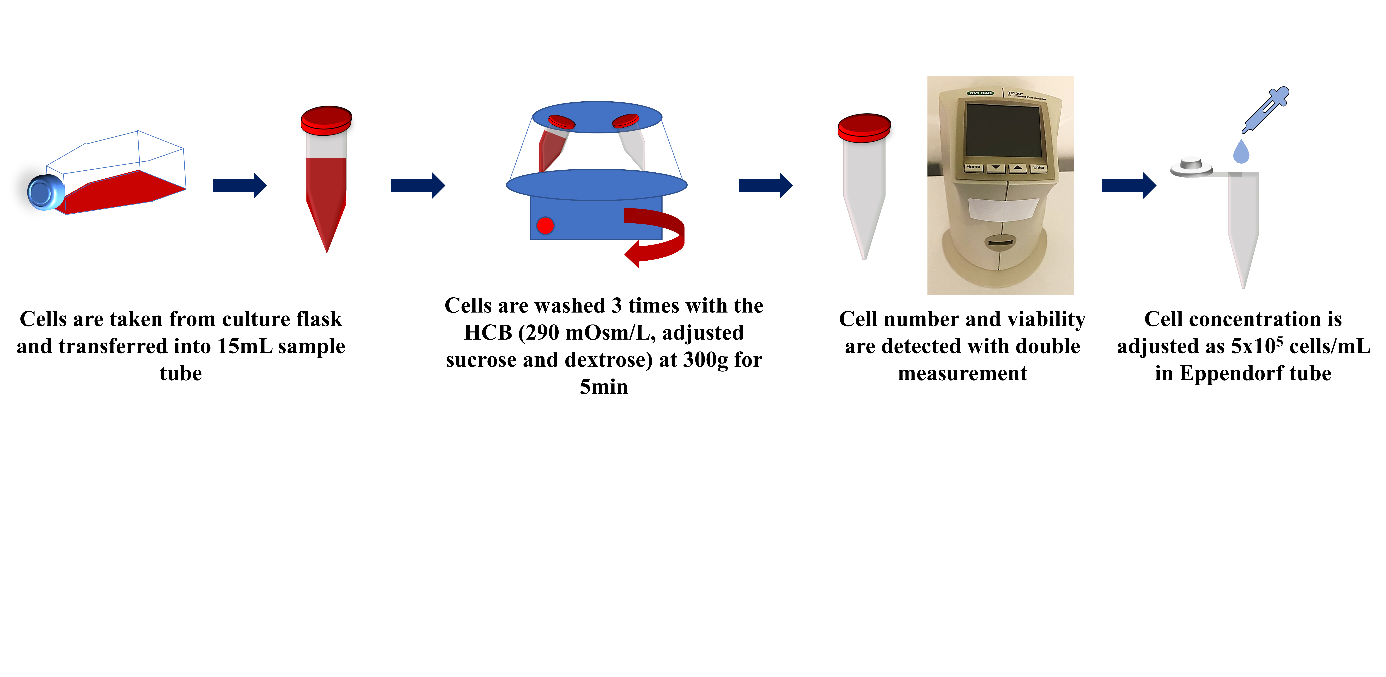


Figure S3: Cell preparation. In order to determine cell number and viability, two different samples from cell population were measured in automated cell counter (as double measurement).

- 1. **Test setup**

Figure S4 presents the test setup.


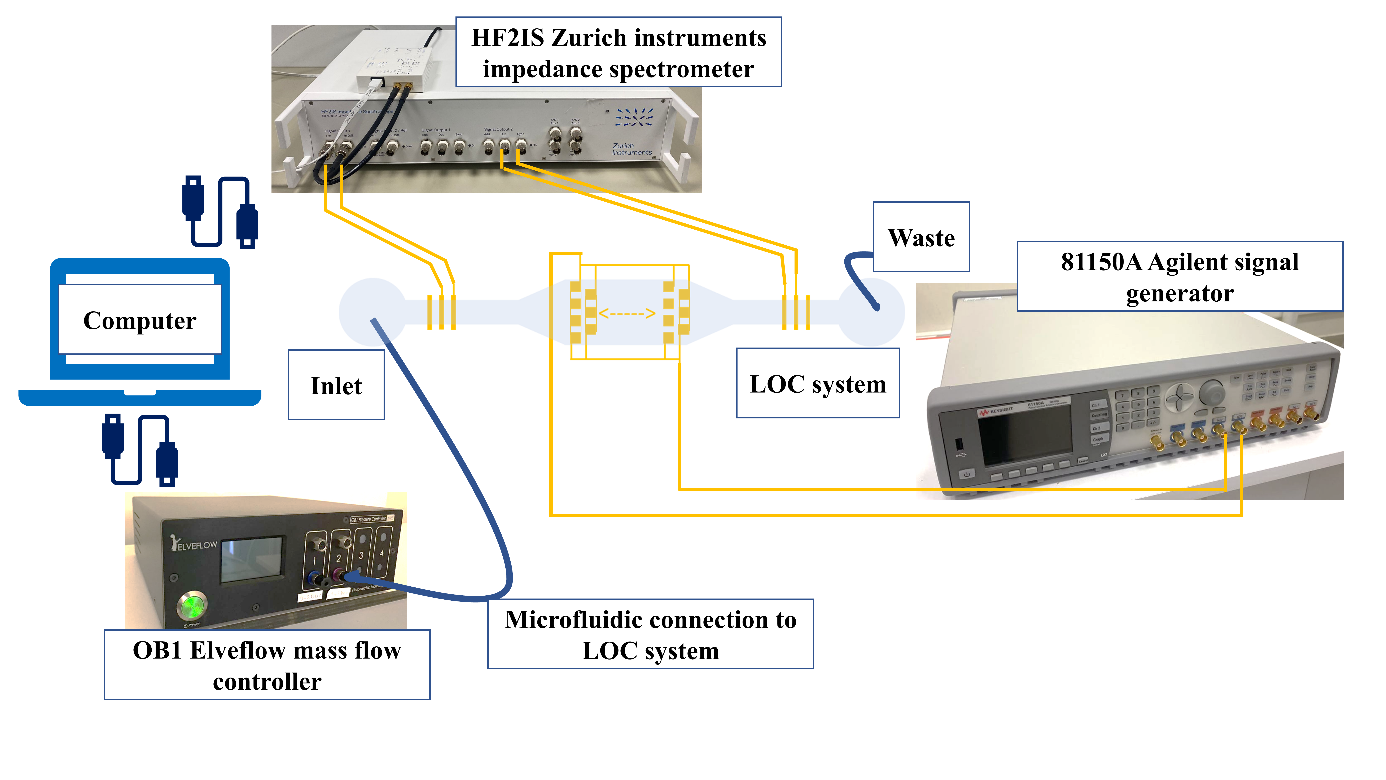


Figure S4: Test setup.

- 1. **Time delay between IM-C units**

The time delay occurred between IM-C units due to cell movement in DEP-D unit, i.e. physical spacing between IM-C units (Fig. S5). This caused misinterpretation of data, since some of the cells were counted in IM-C unit at the entrance of DEP-D unit, stayed in DEP-D region although they were not trapped, and counted as trapped since data recording was stopped. To eliminate this irrelevant data, the time delay caused by cell flow through DEP-D region should be determined. For this purpose, the outputs of IM-C units were cross-correlated via *XCORR* function in Signal processing toolbox of MATLAB.

Figure S5: The application of cross-correlation and determination of time delay (∆t) to obtain accurate differential counting results in IM-C units.

1. **Results**
   1. **DEP force calculation**

The DEP force (Eq. S1) exerted on MDR cells is normalized by the DEP force exerted on wild type cells by assuming the same electric field gradient and neglecting drag force, assumed similar enough for all cells in these analyses, to detect the difference, which is only caused by cell properties (Eq. S5).

| $F_{norm}=\frac{r_{MDR}^{3}{\mathrm{Re}(f_{CM})}_{MDR}}{r_{WT}^{3}{\mathrm{Re}(f_{CM})}_{WT}}$ | (S5) |
| --- | --- |

MDR and WT are used to specify multidrug-resistant and wild type cells in this equation, respectively.

- 1. **Nonspecific cell trapping ratio**

Table S2 presents the nonspecific trapping ratio of cells analyzed in this study. Means of trapping ratios were not significantly different for both K562 and CCRF-CEM cells and their drug-resistant progenies (n=2, p<0.05). Therefore, nonspecific trapping can be neglected.

Table S2: Nonspecific trapping ratio of K562/wt and K562/imaR cells in HCB, having 200 mS/m conductivity, and CCRF-CEM/doxR and CCRF-CEM/wt cells in different DEP media. Measurements are duplicate and results are presented as mean ± standard deviation.

|  | **200 mS/m** | | |
| --- | --- | --- | --- |
| **K562/wt** | 6.0% (±0.2%) | | |
| **K562/imaR** | 2.9% (±4.0%) | | |
|  | **110 mS/m** | **125 mS/m** | **160 mS/m** |
| **CCRF-CEM/wt** | 5.7% (±0.5%) | 2.8% (±3.1%) | 1.2% (±1.7%) |
| **CCRF-CEM/doxR** | 7.9% (±9.7%) | 2.3% (±0.6%) | 7.5% (±6.4%) |

1. **Flow cytometry analysis**

Cells were centrifuged at 300 g for 5 min. Concentration was adjusted as 10^6^ cells/ml with Automated Cell Counter (TC20, BioRad). Fluorescently tagged antibodies (Biolegend, Inc.): Anti CD243-PE, anti MRP1- Alex647, and anti CD338-FITC, were added into cell solutions to label P-gp, MRP1, and BCRP on the membrane, respectively. 45 min. incubation was applied at 4°C. One washing was made to reduce background fluorescence. Finally, cells were analyzed with flow cytometry (BD FACSAria™ III, BD Biosciences) for the comparative expression level of proteins at the same time. Measurements were carried out with Utku Horzum in Hacettepe University. An exemplary measurement set out of duplicated ones is presented in Figure S6.

To eliminate autofluorescence caused by auto fluorescent drugs, such as doxorubicin, the fluorescence of every cell type was compared with its untagged version. This is presented as control in these plots. There is no important difference between untagged K562/wt and K562/imaR in terms of cell count. Therefore, only one control was presented in plots as examples. This is the same for CCRF-CEM cells. On the other hand, in fluorescence fold increase calculations, median values of untagged version of wild type and resistant cells were used, separately.


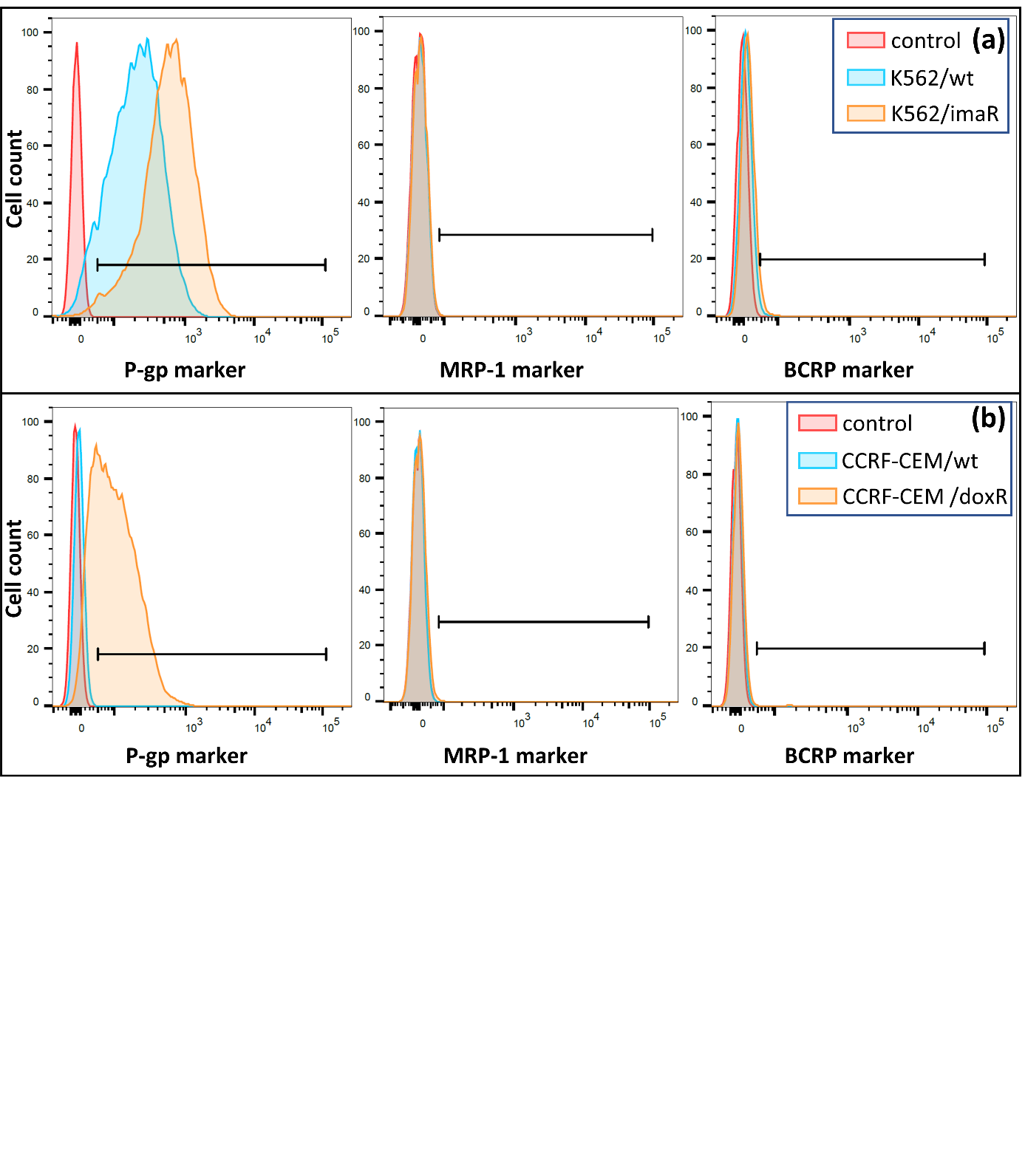


Figure S6: Quantification of P-gp, MRP-1, and BCRP in K562 (a) and CCRF-CEM (b) cells via flow cytometry. An exemplary measurement set out is presented. In control, K562/wt and CCRF-CEM/wt cells were examined via flow cytometry without antibody labeling to analyze autofluorescence of cells.

**References**

1. Pethig, R. How does Dielectrophoresis Differ from Electrophoresis? in *Dielectrophoresis: Theory, Methodology and Biological Applications* 31–47 (John Wiley & Sons, Ltd, 2017).

2. Çetin, B. & Li, D. Dielectrophoresis in microfluidics technology. *Electrophoresis* **32**, 2410–2427 (2011).

3. Jones, T. B. Basic Theory of Dielectrophoresis and Electrorotation. *IEEE Eng. Med. Biol. Mag.* **22**, 33–42 (2003).

4. Labeed, F. H., Coley, H. M., Thomas, H. & Hughes, M. P. Assessment of multidrug resistance reversal using dielectrophoresis and flow cytometry. *Biophys. J.* **85**, 2028–2034 (2003).

5. Demircan Yalçın, Y., Sukas, S., Töral, T. B., Gündüz, U. & Külah, H. Exploring the relationship between cytoplasmic ion content variation and multidrug resistance in cancer cells via ion-release based impedance spectroscopy. *Sensors Actuators, B Chem.* **290**, (2019).
